# A comparison of functional vestibulo-ocular reflex and proprioception in athletes of combat sports and ball sports

**DOI:** 10.1016/j.heliyon.2023.e17540

**Published:** 2023-06-25

**Authors:** Fatma Kızılay, Deniz Uğur Cengiz

**Affiliations:** a Department of Physiotherapy and Rehabilitation, Faculty of Health Sciences, Inonu University, 44280, Malatya, Turkey; b Department of Audiology, Faculty of Health Sciences, Inonu University, 44280, Malatya, Turkey

**Keywords:** Vestibulo-ocular reflex, Proprioception, Combat sports, Ball sports

## Abstract

**Background:**

The contribution and role of sensory systems in providing postural control may vary depending on the condition or activity. Vestibulo-ocular reflex (VOR) and proprioception might be affected by the type of sport.

**Objective:**

This study aimed to compare athletes engaged in combat sports and ball sports in terms of functional VOR and proprioception.

**Design:**

This study is a cross-sectional, comperative research.

**Method:**

Twenty-four athletes engaged in combat sports and 20 in ball sports were included in the study. Functional examination of VOR in athletes was performed with a novel Functional Head Impulse Test (fHIT). Proprioception analysis was evaluated using Laser Pointer Assisted Angle Reproduction Test (LPA-ART) in 90° shoulder flexion and abduction.

**Results:**

fHIT lateral and posterior % Correct Answer (CA) values and anterior SCC 6000°/s^2^ values of athletes engaged in ball sports were found to be statistically significantly higher than athletes in combat sports (p < 0.05). Except for anterior SCC 4000°/s^2^ value, scores of the ball players in all accelerations and directions were higher than combat sports athletes, although it was not statistically significant. The 90° shoulder flexion and abduction angle deviation values obtained from the LI-ATT were statistically lower in those who played ball sports (p < 0.05).

**Conclusions:**

Participation in sports branches played with the ball is expected to provide gains for athletes who want to reach high-level performance in proprioceptive and vestibular functioning and those with vestibular/proprioceptive pathology. Maintaining clear vision in dynamic sports branches can be improved by training VOR function.

## Highlights

• Functional Head Impulse test (fHIT) was used in the evaluation of the vestibulo-ocular reflex (VOR) of athletes who participated in the study.
• VOR function and proprioception vary according to the type of sport.
• Both VOR and proprioception functions are superior in athletes of ball sports compared to athletes of combat sports.
• Participation in ball sports is beneficial for improving VOR function and proprioception in the increasing sportive performance.

## 1 Introduction

Sportive performance gains importance at an increasing and accelerating level from the past to the present due to the increased importance attributed to sportive success in the international arena, the large economic budgets of sports organizations, and increasing competition. Athletes, sports scientists, and experts working in this field have been striving to improve sports performance [1]. The individual factors of the athlete and environmental factors that may affect athletic success are analyzed in detail. Having a healthy sensory-perception-motor function harmony, which is the foremost among them, is accepted as one of the main indicators for optimum performance [1,2]. Even if there is a minimal deficiency concerning the sense organs in the learning, development, and maintenance of sportive movements, this condition may cause negative consequences on a large scale. It is known that sensory stimuli and feedback mechanisms are provided by visual, vestibular, proprioceptive, and tactile systems [3]. The contribution and role of sensory systems in providing postural control may vary depending on the condition or activity. It was reported that the more active/dominant sensory information for a particular situation assumes a greater role [4]. Among these sensory systems, the most easily observed contribution is the sense of sight, since opening and closing the eyes can be easily compared in tests [5]. In terms of the importance of vision in sports, it is emphasized that features such as static vision, dynamic visual acuity, and the cooperation of the eyes and extremities during movements should also be trained [5]. Providing visual perception during head movements is related to the function of the vestibulo-ocular reflex (VOR) system [6]. To this end, it is interesting to evaluate the function of the VOR system, which makes a significant contribution to visual acuity, especially in some ball-oriented sports that require dynamic head movements. Head movements, upper extremity, and eye perception are expected to work in good coordination for requirements such as ball tracking and correct handling of the ball. In branches such as combat sports, which need skills related to relatively less dynamic head and eye movements, there is not enough evidence in the literature to differentiate these functions from sports where dynamic head movements are used more frequently, requiring ball tracking. The literature does not provide sufficient evidence to answer the question of which sports branch the functional VOR will contribute more.

In recent years, the importance of proprioceptive sense in improving sports performance and returning to sports after injuries have been realized more [7]. Proprioception is among the somatosensory senses. It is known that the proprioceptive sense performs functions in body awareness, spatial positioning, and maintenance of movement [8]. It has been reported that the development of proprioceptive functions is also of great importance in postural control mechanisms, protection from injuries, and revealing general sportive performance [5,9].

This study was planned based on the hypothesis that the proprioceptive function and the functional VOR system can contribute to sportive performance, and therefore, it was planned to determine the level of this contribution in terms of sports branches. It has been reported in studies that proprioceptive functions, visual skills, and dynamic visual acuity, which are known to be directly related to VOR function, are better in athletes [10,11]. However, there exists no study on the comparison of functional VOR and proprioceptive function between sports branches. At this point, it was aimed to compare the athletes in basketball, volleyball, and handball, which are played with focus on ball, and the athletes who are engaged in combat sports in terms of functional VOR and proprioception.

## 2 Materials and methods

### 2.1 Variables

In the research, sports branch is the independent variable, and VOR and proprioception are the dependent variables examined in sports branches.

### 2.2 Study design and setting

The research was designed in a methodological cross-sectional study. The study was single-centered. The data collection phase of the research was carried out between April 15, 2022 and August 15, 2022 on students who were engaged in ball sports and combat sports at Inonu University Faculty of Sports Sciences.

### 2.3 Participants

The study was carried out on instutional athletes aged 18–35 playing basketball, volleyball, handball, taekwondo, judo, or wrestling. There were a total of 72 athletes from these branches in the research center. However, a total of 51 athletes volunteered to participate in the study.

### 2.4 Inclusion/exclusion criteria of the research

These individuals were included in the study; athletes who have been playing ball sports regularly for at least 6 months and who have been involved in one of the branches of combat sports were included in the study.

These individuals were excluded; (1) involved in more than one main sports branch; or (2) had a surgical operation in the previous 1 year or a sports injury in the previous 6 month, or; (3) had a permanent disease affecting visual function (n = 2). Participants who have not met the inclusion criteria, who could not complete the required tests during the study, and who quitted the study were excluded (n = 5).

**Fig. 1:**
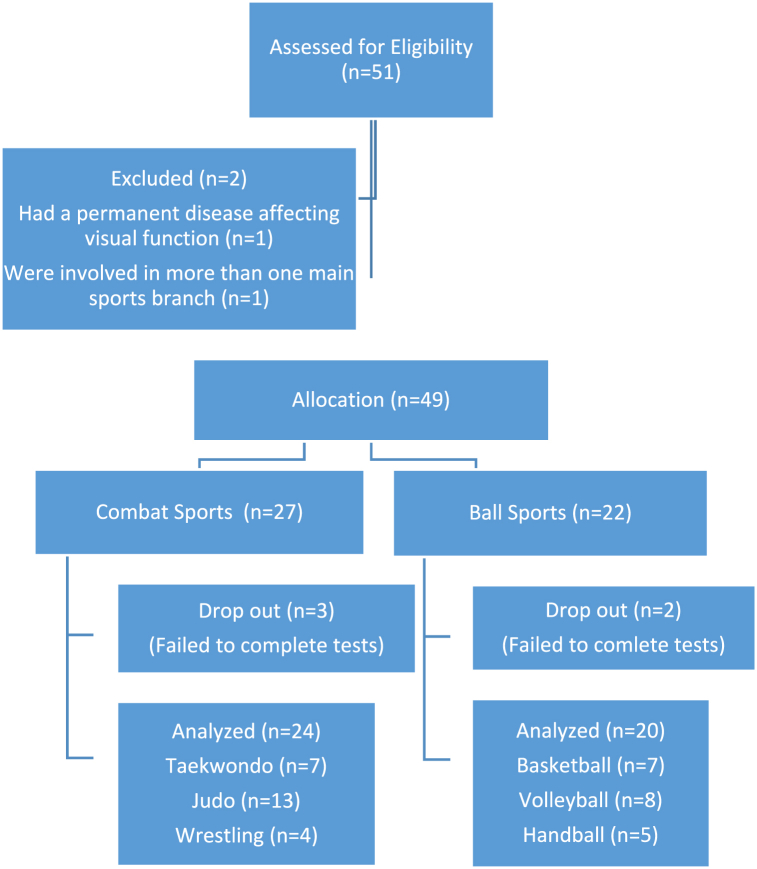
Flow chart of the participants.

As a result, the results of 44 athletes were analyzed. The distribution of the number of athletes according to the branches was basketball (n = 7), volleyball (n = 8), handball (n = 5), taekwondo (n = 7), judo (n = 13), wrestling (n = 4). The flow chart of the participants is presented in Fig. 1.

### Ethics approval

Written permission was obtained from the Inonu University Faculty of Sports Sciences and ethical approval was obtained from Inonu University Health Sciences Non-Invasive Clinical Research Ethics Committee (2022/3275). The study was conducted by the principles of the Declaration of Helsinki. Volunteers were informed about the purpose and content of the study and their written consent was signed.

### 2.5 Data sources/measurement

#### 2.5.1 Sociodemographic and sport spesific characteristics

A case report form in physical format was prepared by the researchers in order to question the demographic information of the participants and their participation in sports, and it was applied to the participants in the initial stage of the research procedure. In the form, sociodemographic characteristics (such as age, gender, marital status, education level, concomitant diseases, medications used (if any), history of previous surgery of the participants) were questioned with 16 questions, and sport-specific features (sports branch, duration of engaging in sports (months), frequency of engaging in sports (days/week) were questioned with 3 questions. A stadiometer with an accuracy of 0.1 m (Seca 213) was used to measure height of participants. Body weight of the participants were measured using an electronic scale (Tanita TBF 401 A, Japan) with an accuracy of ±100gr. The resulting value was recorded in kg. Body Mass Index (BMI) calculated using the formula weight/height [2] (kg/m^2^).

#### 2.5.2 Functional Head Impulse test (fHIT)

Functional evaluation of the VOR has been performed with the BEON Solutions srl (Zero Branco, Italy) fHIT device. fHIT is a special test technique that evaluates visual acuity during head movements. It works according to the principle of correct reading of the Landolt C character displayed on the screen during the accelerations (impulses) given to the participant's head by the researcher applying the test. The fHIT consists of a computer, a gyroscope, a mini keyboard, and software. Individuals are seated at a distance of 1.5 m from the computer monitor and their static vision is evaluated first. This evaluation was made by showing 8 different Landolt C characters from the largest size to the smallest size and the participant selects the corresponding character they can see by hitting the relevant key on the mini keyboard. According to the result, the next phase in which head impulses will be made is started. The individual is asked to key in the mini keyboard in accordance with the Landolt C shapes seen on the screen by swinging their head in lateral (4000–6000°/s^2^) and posterior and anterior (3000–6000°/s^2^) semicircular canal (SCC) planes. With the fHIT, frequency-specific and plane-induced percentage correct answer (%CA) evaluations are made in each plane [12,13].

#### 2.5.3 Upper extremity proprioception evaluation

The Laser Pointer Assisted Angle Reproduction Test (LPA-ART) was applied for Joint position sense (JPS) evaluation. LPA-ART test is applied in two ways with eyes open and closed. It is based on the calculation of the angular deviation between the 2 tests by the participant in the open and closed eye positions. In order to prevent the laser pointer from slipping and being affected by wrist movements, it was fixed on the athlete's wrist with the help of a strip. Participants were asked to keep the elbow in full extension. In proprioceptive evaluation, measurements were made at 90° for flexion and abduction movements of shoulder joint for JPS. A grid background (mm) fixed to the wall was used in the LPA-ART setup. The 90° shoulder flexion and abduction positions of the person were measured using a goniometer. During the LPA-ART, the points indicated by the laser cursor were marked while the eyes were open and then closed. The first marking on grid background was made with eyes open in the participant's upper extremity position as measured by the researcher. Afterwards, the participant blindly repeated 3 tests and the best score was marked. The angular deviation between the 2 marked points was calculated in 'degrees' (°). Calculation was made according to the TAN 35° (opposite/adjacent) formula to evaluate angular deviations [14]. The LPA-ART was performed in the dominant extremity of all participants. The LPA-ART was developed in 2011 by Balke et al. [15]. In the evaluation of proprioception based on joint position sense, the LPA-ART is frequently used in clinics and research because it is a practical, easy-to-apply, and economical tool [14,16].

### 2.6 Bias

The performance data of the study (VOR function analysis and proprioception analysis) and the demographic data of the athletes participating in the study were collected by two different researchers. The researcher who collected the performance data was blind to participants’ information about sport branches.

### 2.7 Study size

In the power analysis to determine the sample size, the type I error was 0.05 and the type II error was 0.2. The power of the test was determined as 0.8. In the analysis, based on the study of Corallo et al. [17], it was calculated that the minimum number of volunteers to be included in each group should be 12 to obtain a significant difference of 0.2182 units in two different groups in the vHIT (Video Head Impulse Test) score [17]. Power analysis was performed using the G-Power 3.1.7 package program (Heinrich-Heine-Universität, Dusseldorf, Germany).

### 2.8 Statistical analysis

The analysis of the data included in the research was carried out with the SPSS-25 (Statistical Program in Social Sciences- IBM, Armonk, NY, USA) package program. The Kolmogorov-Smirnov Test was used to check whether the data included in the study fit the normal distribution [18]. The significance level (p) for comparison tests was set as 0.05. Since the variables did not have a normal distribution (p > 0.05), the analysis was made with non-parametric test methods. In comparisons in independent pairs, since the assumption of normality was not provided, the Mann-Whitney *U* test was used. In the analysis of categorical data, contingency tables were created and the Chi-square (ꭓ2) analysis was performed. Spearman rank correlation coefficient was used in the relationships between numerical variables.

## 3 Results

The distribution of the participating athletes by gender, educational status and sports branches is given; it was tested whether the participants included in the study differences between the groups according to demographic variables and the results are given in Table 1.

**Table 1:** Comparison of groups by distribution of demographic variables.

Variable	Group		Combat Sports (n = 24)	Ball Sports (n = 20)	Total	ꭓ^2^	p^a^
**Gender**	**Female**	**n**	6	7	13	−0.109	0.469
		**%**	25.0%	35.0%	29.5%		
	**Male**	**n**	18	13	31		
		**%**	75.0%	65.0%	70.5%		
**Education Status**	**Undergraduate**	**n**	24	20	44	–	–
		**%**	100.0%	100.0%	100.0%		
**Sports Branch**	**Taekwondo**	**n**	7	–	7	1	0.001*
		**%**	29.2%	–	15.9%		
	**Judo**	**n**	13	–	13		
		**%**	54.2%	–	29.5%		
	**Wrestling**	**n**	4	–	4		
		**%**	16.6%	–	9.1%		
	**Basketball**	**n**	–	7	7		
		**%**	–	35.0%	15.9%		
	**Volleyball**	**n**	–	8	8		
		**%**	–	40.0%	18.2%		
	**Handball**	**n**	–	5	5		
		**%**	–	25.0%	11.4%		
**Variable**			**Combat Sports (n = 24)**	**Ball Sports (n = 20)**	**Mann Whitney-U**		**p**^**b**^
**Age (Year)**		**Mean ± sd**	21.3 ± 1.6	21.3 ± 1.8	238.500		0.971
		**M (Min - Max)**	21 (19–25)	21 (18–26)			
**BMI (kg/m**^**2**^**)**		**Mean ± sd**	22.8 ± 2.9	22.2 ± 2.7	202.000		0.370
		**M (Min - Max)**	23.5 (17.0–29.4)	21.5 (18.4–28.7)			
**Time to Participate in Sports (Year)**		**Mean ± sd**	5.4 ± 3.3	6.3 ± 3.8	209.000		0.462
		**M (Min - Max)**	4.5 (1–12)	6 (1–13)			
**Frequency of Participate in Sports (Day/Week)**		**Mean ± sd**	3.8 ± 0.8	3.9 ± 1.4	227.500		0.748
		**M (Min - Max)**	4 (3–5)	3 (3–7)			

n; number of samples, %; percent, pa; Chi-square Test value (ꭓ^2^), SD; Standard Deviation, p^b^; Mann Whitney *U* test, p value, *p < 0.05; there is a statistically significant difference between the groups, BMI: Body Mass Index.

No statistically significant difference was found between athletes engaged in ball sports and those engaged in combat sports, according to age, education status, sports branch, BMI, duration and frequency of engagement in sports, and gender variables (p > 0.05, Table 1). The groups showed a homogeneous distribution according to age, education, branch, BMI, duration and frequency of engagement in sports, and gender.

A statistically significant difference was found between ball sports players and combat sports athletes in terms of the amount of angular deviation (◦) in proprioception at 90° shoulder flexion and 90° shoulder abduction measurements (p < 0.05). In terms of angular deviation values; Combat sports athletes had higher deviation scores of 2.8° in the measuremet of shoulder 90° flexion and 1.9° in the shoulder 90° abduction compared to the ball sports athletes (Table 2).

**Table 2:** Comparison of VOR and proprioception variables between groups.

Measurements	Groups	Mean ± sd	M (Min-Max)	Test	p
**Proprioception**	**Combat Sports**	7.1 ± 2.5	7.5 (2.5–12)	***97.500***	0.001*
**90° flexion (**^**0**^**)**	**Ball Sports**	4.3 ± 2.1	3.5 (2–11.5)		
**Proprioception**	**Combat Sports**	6.1 ± 2.3	6 (2.5–11.5)	***102.500***	0.001*
**90° abduction (**^**0**^**)**	**Ball Sports**	4.2 ± 1.6	3.7 (2–10)		

Sd; standard deviation. Min; lowest score. max; highest score. test value; Mann Whitney Test Value. p value; statistical significance. *p < 0.05; There is a statistically significant difference between the groups. ◦: Degrees.

The difference between ball sports players and combat sports athletes in the measurement of lateral %CA, posterior %CA, and anterior 6000/s^2^ scores was found statistically significant (p < 0.05). In combat sports and ball sports athletes, the difference in lateral %CA mean scores was 4.4 points, posterior %CA difference was 4.2 points, anterior 6000°/s^2^ difference was 7.5 points in favor of ball sports players, and these differences were statistically significant (Table 3).

**Table 3:** Intergroup comparison of fHIT SSC measurements.

Measurements	Groups	Mean ± sd	M (Min-Max)	Test	p
**Lateral 4000°/s**^**2**^	**Combat Sports**	93.9 ± 7.8	100 (75–100)	172.000	0.057
	**Ball Sports**	97.9 ± 4.4	100 (87.5–100)		
**Lateral 5000°/s**^**2**^	**Combat Sports**	88.8 ± 10.6	88.7 (68–100)	209.000	0.457
	**Ball Sports**	91.3 ± 7.0	91.5 (75–100)		
**Lateral 6000°/s**^**2**^	**Combat Sports**	83.6 ± 13.4	87.5 (50–100)	163.000	0.063
	**Ball Sports**	91.2 ± 9.3	90 (75–100)		
**Lateral %CA**	**Combat Sports**	89.3 ± 5.8	89.5 (78.3–100)	***138.500***	0.016*
	**Ball Sports**	93.7 ± 4.7	92.7 (86.6–100)		
**Posterior 3000°/s**^**2**^	**Combat Sports**	94.7 ± 10.5	100 (62.5–100)	221.500	0.552
	**Ball Sports**	97.3 ± 5.7	100 (83.5–100)		
**Posterior 4000°/s**^**2**^	**Combat Sports**	91.8 ± 8.1	92.2 (75–100)	194.500	0.255
	**Ball Sports**	94.3 ± 8.2	100 (75–100)		
**Posterior 5000°/s**^**2**^	**Combat Sports**	87.9 ± 10.7	90.7 (67.5–100)	167.000	0.074
	**Ball Sports**	93.3 ± 10.0	100 (66.5–100)		
**Posterior 6000°/s**^**2**^	**Combat Sports**	86.6 ± 11.9	85.5 (65–100)	173.000	0.092
	**Ball Sports**	91.9 ± 13.2	100 (46–100)		
**Posterior %CA**	**Combat Sports**	89.9 ± 6.3	89.8 (74.2–100)	***135.500***	0.013*
	**Ball Sports**	94.1 ± 7.5	97.1 (69.6–100)		
**Anterior 3000°/s**^**2**^	**Combat Sports**	96.6 ± 7.9	100 (67–100)	225.500	0.611
	**Ball Sports**	98.2 ± 5.1	100 (83.5–100)		
**Anterior 4000°/s**^**2**^	**Combat Sports**	95.5 ± 8.9	100 (70–100)	212.000	0.424
	**Ball Sports**	94.8 ± 7.8	100 (73.5–100)		
**Anterior 5000°/s**^**2**^	**Combat Sports**	91.7 ± 10.3	97.5 (62.5–100)	174.000	0.081
	**Ball Sports**	96.9 ± 5.4	100 (81.5–100)		
**Anterior 6000°/s**^**2**^	**Combat Sports**	88.7 ± 11.3	86.7 (67–100)	***145.000***	0.010*
	**Ball Sports**	96.2 ± 9.7	100 (67–100)		
**Anterior %CA**	**Combat Sports**	92.9 ± 6.4	93.6 (78.5–100)	164.000	0.068
	**Ball Sports**	96.5 ± 4.0	97.3 (86.4–100)		

Sd; standard deviation. Min; lowest score. max; highest score. test value; Mann Whitney Test Value. p value; statistical significance. *p < 0.05; There is a statistically significant difference between the groups. %CA: Percentage of correct answers. ◦: Degrees. s: Seconds.

No statistically significant difference was found between those who play ball sports and those engaged in combat sports in the lateral 4000°/s^2^, 5000°/s^2^, 6000°/s^2^, posterior 3000°/s^2^, 4000°/s^2^, 5000°/s^2^, 6000°/s^2^, anterior 3000°/s^2^, 4000°/s^2^, 5000°/s^2^ and anterior %CA measurement (p > 0.05, Table 3).

## 4 Discussion

In this study, athletes engaged in combat sports and ball sports, such as basketball, volleyball, and handball were compared in terms of functional VOR and proprioception. The importance of vision-related functions in the measurement of other motor skills in the analysis of sportive performance can be easily demonstrated due to the differences in performing the tests with eyes open and closed [5]. It was reported that visual function significantly affects sportive performance, especially in demonstrating motor features such as balance coordination and reaction time [19]. Due to VOR functions, clear vision is preserved during head movements in our daily living activities. During head movements, semicircular canals (SCCs) can be stimulated in the lateral, posterior and anterior planes with a frequency of approximately 0.5–5 kHz and a speed range of 550°/s^2^ - 6000°/s^2^ [20]. Developments in the evaluation of VOR accelerated with the introduction of the fHIT, which was first introduced in 2013 as a new tool for functional testing of the VOR [13], and its importance in functional testing of the vestibular system during head movements we use in daily life has been demonstrated by recent studies [21]. A group of researchers tested the vestibulo-ocular system function using the Video Head Impulse test (vHIT) and fHIT and analyzed dynamic visual acuity in 27 optic neuritis patients. As a result of the study, both vHIT and fHIT examinations correctly classified all patients as abnormal, and more specifically, the fHIT detected more abnormalities than the vHIT [22]. This study demonstrates the specificity of fHIT in functional VOR assessment. In a study researchers examined the change in fHIT scores according to age in a healthy population. In the 18–35 age group, mean fHIT mean scores were 88.46 ± 12.07 for lateral SCC (4000–6000°/s^2^) %CA; 88.76 ± 11.37 for anterior SCC (3000–6000°/s^2^) %CA; and, 90.54 ± 12.19 for posterior SCC (3000–6000°/s^2^) %CA [21]. In current study, while the Lateral %CA was 93.68 ± 4.72 in the ball sports players in the same age group, it was 89.29 ± 5.76 in the combat athletes; posterior %CA was 89.89 ± 6.34 in combat athletes and 94.09 ± 7.5 in ball sports players, and anterior %CA was 92.96 ± 6.41 in combat athletes and 96.47 ± 4.02 in ball sports players. According to these results, it was observed that the lateral, posterior, and anterior %CA values measured in the athletes engaged in combat sports and non-athletes were quite similar. However, the same parameter measurements of the athletes who play ball sports were considerably higher than the values obtained from healthy people aged 18–35 years [21]. According to these results, it is seen that the lateral, posterior, and anterior (which is numerically higher, although not statistically significant) %CA values were obtained in a very high percentage in ball sports players, compared to both the healthy population not engaged in sports and the population of athletes engaged in combat sports. Considering the importance of %CA value in the functional evaluation of VOR, these comments may indicate that being involved in ball sports provides significant gains in terms of VOR function. In another study, researchers established the normative values of the fHIT in an athlete population. In the study, 268 active professional athletes (age: 23.70 ± 5.32 years) from six sports branches (American football, football, handball, ice Hockey, bob and skeleton, snowboard) were tested using the fHIT. According to the results of the study, it was reported that the branch involved in sports had a great effect on the result of the fHIT, in other words, on the functional VOR. It was determined that handball players achieved a better performance than the whole group of athletes, regardless of the direction of head impulses, and the %CA value obtained by the snowboard and bob, and skeleton athletes was significantly lower than that of the entire group of athletes [23]. In our study, although there were differences in the branches investigated, the difference of fHIT in sports branches was investigated similarly to the study. According to our study results, the difference between lateral %CA, posterior %CA and anterior 6000/s^2^ results between the ball sports players (volleyball, basketball, and handball) and the combat sports (taekwondo, judo, and wrestling) athletes was found to be significant in favor of the ball sports (p < 0.05). The present results were in line with the results of the study [21].

It is assumed that the proprioceptive ability specific to the movement difficulties of a sport is related to both the sport-specific training and the fitness level reached by the athlete [24]. While Sherrington, defined proprioception as stimuli coming from deep receptors in 1906 [25], Evarts emphasized that VOR is a natural proprioceptive reflex [26]. We think that this sense, which has been known for over a century, still has more aspects to be explored. Hikita and Kasai, reported that while in a steady state, eye movements during dynamic head movement and adaptation to movement speed are produced by incorporating otolith and extremity proprioceptive signals linearly to visual memory [27]. A group of researchers stated that proprioceptive high-level skill is significantly related to the performance level achieved by elite athletes [11]. In another study, it was reported that proprioceptive training provided significant improvements in balance and performance measurements in futsal players with ankle injuries [28]. In the present study, we evaluated proprioception in the dominant upper extremity of the participants using the LPA-ART. According to our results, in both 90° flexion and 90° abduction angle repetition, the angle deviations of the athletes engaged in ball sports were found to be much lower than the athletes engaged in combat sports. The magnitude of the LPA-ART angle deviation is interpreted as a weak joint position sense [15]. Therefore, we can interpret that engaging in ball sports can be beneficial to improve upper extremity proprioception.

## 5 Conclusions

High-level sportive performance in athletes can be demonstrated by the harmony of the visual, proprioceptive, and vestibular systems. The proprioceptive ability specific to a sport branch is related to the training applied and the level of fitness achieved. The issue of vestibular function can be analyzed with the help of current and objective methods such as the fHIT. According to the study outcomes, the type of sports branch is effective on functional VOR and proprioception. It is predicted that playing ball sports provides positive gains both for athletes who want to reach high-level performance in terms of proprioceptive and vestibular vantage points and for people with vestibular or proprioceptive pathology. It is considered that encouraging participation in sports branches suitable for vestibular rehabilitation programs in the treatment of these pathologies will positively affect the process. In the foreseeable future, the development of specific skills such as proprioception and vestibular functions will become more important in terms of athletic performance. Although the question in which sports branches these skills are more developed can be partially revealed by our study, it is clear that there is a need for studies with wider participation from different sports branches.

Due to the fact that the study was carried out in a single center, the study was carried out on a small sample size. This is an important limitation of the study. Because the study was conducted in a single center, the results cannot be generalized to the entire population. Multicenter studies are needed. The study presents the results of a limited number of athletes from a limited number of sports branches. In future studies, there is a need for studies in which participants will be examined from many different sports branches such as football, swimming and athletism.

## Author contribution statement

Fatma Kızılay, Deniz Uğur Cengiz: Conceived and designed the experiments; Performed the experiments; Analyzed and interpreted the data; Contributed re-agents, materials, analysis tools or data; Wrote the paper. Fatma Kızılay: Analyzed and interpreted the data; Contributed reagents, materials, analysis tools or data; Wrote the paper.

## Funding statement

This research did not receive any specific grant from funding agencies in the public, commercial, or not-for-profit sectors.

## Data availability statement

Data included in article/supplementary material/referenced in article.

## Declaration of competing interest

The authors declare that they have no known competing financial interests or personal relationships that could have appeared to influence the work reported in this paper.
